# PTCH-1 and MDM2 expression in ameloblastoma from a West African sub-population: implication for chemotherapeutics

**DOI:** 10.11604/pamj.2015.20.140.5869

**Published:** 2015-02-17

**Authors:** Samuel Ebele Udeabor, Akinyele Olumuyiwa Adisa, Ahmed Oluwatoyin Lawal, Mike Barbeck, Patrick Booms, Robert Alexander Sader, Shahram Ghanaati

**Affiliations:** 1 Department of Oral and Maxillofacial Surgery, College of Health Sciences, University of Port Harcourt, Nigeria; 2 Department of Oral Pathology, College of Medicine, University of Ibadan, Nigeria; 3 Department for Oral, Cranio-Maxillofacial and Facial Plastic Surgery, Medical Center of the Goethe University Frankfurt, Frankfurt am Main, Germany; 4 REPAIR-Lab, Institute of Pathology, University Medical Center, Johannes Gutenberg University, Mainz, Germany

**Keywords:** PTCH-1, MdM2, ameloblastoma, chemotherapeutics

## Abstract

**Introduction:**

Ameloblastoma is a slow growing, painless odontogenic swelling which can attain sizes that result in severe deformities of the craniofacial complex. It is the most commonly encountered odontogenic tumor in Nigeria. Surgical intervention is currently the method of treatment; however identification of altered molecular pathways may inform chemotherapeutic potential. The Protein Patched homolog 1 (PTCH-1) is overexpressed in ameloblastoma. Also, mutation in the MDM2 gene can reduce the tumor suppressor function of p53 and promote ameloblastoma growth. No study however has characterized the molecular profile of African cases of ameloblastoma with a view to developing chemotherapeutic alternatives. The objective was to characterize the PTCH-1 genetic profile of Ameloblastoma in Nigerian patients as a first step in investigating its potential for chemotherapeutic intervention.

**Methods:**

Twenty-eight FFPE blocks of ameloblastoma cases from Nigerian patients were prepared for antibody processing to PTCH-1 (Polyclonal Anti-PTCH antibody ab39266) and MDM2 (Monoclonal Anti-MDM2 antibody (2A10) ab16895). Cytoplasmic brown staining was considered as positive for PTCH while nuclear staining was positive for MDM2.

**Results:**

Moderate and strong expressions for PTCH in ameloblast and stellate reticulum were 78.6% and 60.7% respectively. Only 3 (10.7%) cases expressed MDM2.

**Conclusion:**

The importance of our study is that it supports, in theory, anti-PTCH/SHH chemotherapeutics for Nigerian ameloblastoma cases and also infers the possible additional use of anti-p53 agents.

## Introduction

Ameloblastoma is a slow growing, painless odontogenic neoplasm which causes expansion of the buccal and lingual jaw plates and sometimes infiltration of soft tissue. Although it is slow growing, the tumor can attain sizes that result in severe deformities of the craniofacial complex [1]. Ameloblastoma of the jaws is the most commonly encountered odontogenic tumor in Africa [2-4]. A Nigerian study reported that ameloblastoma was the commonest odontogenic tumor and constituted 63% of odontogenic tumors [4]. Surgical intervention is currently the main method of treatment and although chemotherapy has been proposed, it is not yet a conventional method of treating ameloblastoma [1]. The identification of altered molecular signaling pathways [5, 6] however, may inform potential nonsurgical approaches of management.

The Sonic Hedgehog (SHH) pathway has members that play a critical role in tooth development, are involved in odontogenic tumorigenesis and induce or promote carcinogenesis in organs [7-9]. Protein Patched homolog 1 (PTCH-1), a tumor suppressor protein, is one of such members. DeVilliers et al [10] identified the overexpression of PTCH-1 in all ameloblastoma cases they studied. The high expression of PTCH is important because certain therapeutic compounds have proven effective as antagonists of the PTCH in SHH pathway. Cyclopamine for example, acts at the level of SHH signaling and is effective in reducing the viability of cancer cells by blocking activation of the SHH response pathway and abnormal cell growth. Studies have shown the effectiveness of cyclopamine on cells of breast cancer, gastric cancer, medulloblastoma and oral squamous cell carcinoma [11, 12]. No study however has characterized the molecular profile of African cases of ameloblastoma with a view to developing therapeutic alternatives to surgery. The p53-MDM2 paradigm is the best-studied relationship between a tumor suppressor gene and an oncogene. These two genes form an auto-regulatory feedback loop in which p53 positively regulates MDM2 levels and MDM2 in-turn negatively regulates p53 by inhibiting its functions as a transcription factor and also inducing intracellular p53 degradation [13]. Therefore a mutation in the MDM2 gene promoter can enhance MDM2 expression and consequently reduce the tumor suppressor function of p53 [14]. MDM2 overexpression has been reported in ameloblastoma and hence linked to tumor progression [15]. We aim to describe PTCH-1 and MDM2 expression in ameloblastoma from Nigerian patients to characterize the possible direct or implied chemotherapeutic potential presented by PTCH-1 and p53 in this neoplasm.

## Methods

Twenty-eight FFPE blocks of ameloblastoma cases from the Oral Pathology Department of the University College Hospital and University of Ibadan, Nigeria were sectioned and stained with hematoxylin and eosin for re-evaluation and inclusion. At the REPAIR laboratory, Institute of Pathology, School of Medicine, University of Mainz Germany, sections were prepared for antibody processing to PTCH-1 (Polyclonal Anti-PTCH antibody ab39266) and MDM2 (Monoclonal Anti-MDM2 antibody (2A10) ab16895) for each specimen, using the specification of the manufacturers. The sections were de-paraffinized, hydrated and then rinsed in phosphate-buffered solution (PBS). They were immersed in heat-induced epitope retrieval citrate buffer of concentration 15mMol and pH 6.0, diluted 1:10 with distilled water and incubated at 95 0C for 10 minutes. They were then placed in fresh citrate, cooled in water for 20 minutes and then rinsed in PBS for 6 minutes. Positive controls in the manufacturers pack were exposed like the test cases. For negative control, slide section produced from each paraffin block was incubated with PBS only, instead of the primary antibodies. All positive and negative controls followed the same laboratory protocol for immunohistochemical staining as the other slides to ensure validity. Envision FLEX peroxidase blocking reagent was added to each section for 5 minutes, and the sections were rinsed in 0.1% PBS for 6 minutes. The specimen were incubated for 30 minutes with 1:100 dilutions of Abcam Rabbit Polyclonal Anti-PTCH antibody ab39266 and Abcam Mouse Monoclonal Anti-MDM2 antibody (2A10) ab16895, then rinsed with PBS, followed by incubation with undiluted EnVision FLEX/Horse Radish Peroxidase for 20 minutes. One ml of diaminobenzidine solution was added to cover the specimen, followed by incubation in a humidity chamber for 15 minutes. The sections were then immersed in aqueous Meyer's haematoxylin and rinsed in distilled water for 5 minutes. The tissue was dehydrated and subsequently rinsed with xylene. Distyrene plasticizer in xylene mounting fluid was then applied, and a cover slip placed.

Cytoplasmic brown staining was considered as positive for PTCH while nuclear staining was positive for MDM2. The Sinicrope [16] scoring method was used to evaluate both the intensity of the immunohistochemical staining and the proportion of the stained epithelial cells. The staining intensity was classified as weak, moderate, or strong. The positive cells were quantified as a percentage of the total number of epithelial cells and assigned to one of five categories (0, <5%; 1, 5-25%; 2, 26-50%; 3, 51-75%; 4, > 75%). The percentage of positivity of the tumor cells and the staining intensities were then multiplied in order to generate an immuno-reactive score. The product of the proportion and intensity scores were calculated such that a final score of 0 indicated no expression, 0-4 indicated weak expression, 5-8 indicated moderate expression and 9-12 indicated strong expression. Each sample was examined and scored by an oral pathologist. The data were analyzed by descriptive statistics using version 20 of the SPSS IBM Corp.

## Results

**Figure 1:**
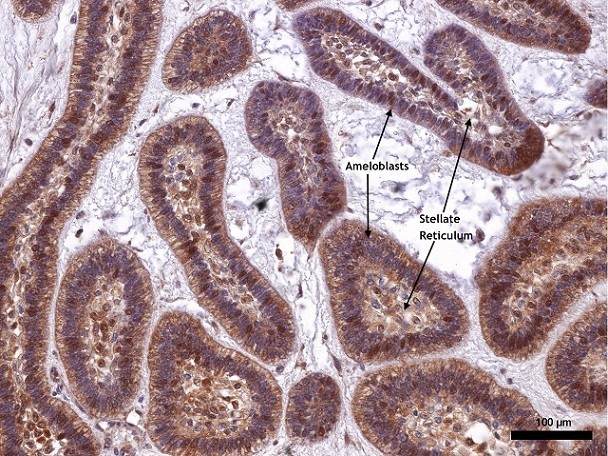
Showing odontogenic epithelial islands descriptive of a follicular ameloblastoma. The peripherally palisaded ameloblast with reversed polarity have +++ staining intensity, while the central stellate reticulum-like area have a ++ staining intensity for PTCH (X100). The proportion of positively stained cells is >75% of neoplastic cells

**Figure 2:**
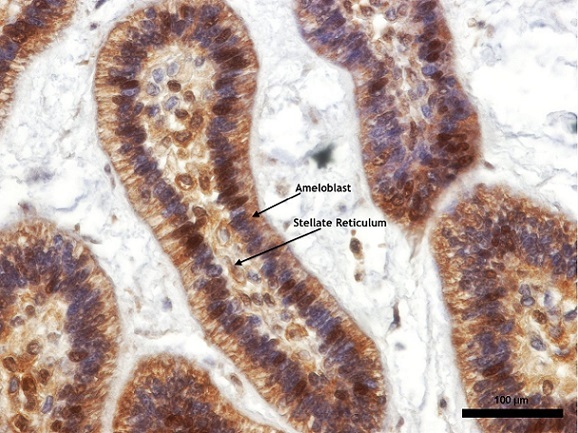
Showing odontogenic epithelial islands descriptive of a follicular ameloblastoma. The peripherally palisaded ameloblast with reversed polarity have +++ staining intensity, while the central stellate reticulum-like area have a ++ staining intensity for PTCH (X200)

**Figure 3:**
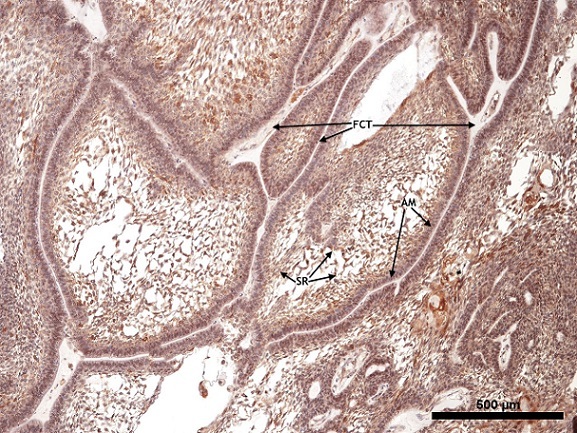
Showing odontogenic epithelial islands descriptive of plexiform ameloblastoma. The peripherally palisaded ameloblast (AM) with reversed polarity have +++ staining intensity, while the central stellate reticulum-like area (SR) have a ++ staining intensity for PTCH (X40). The fibrous connective tissue (FCT) is thinned out

**Figure 4:**
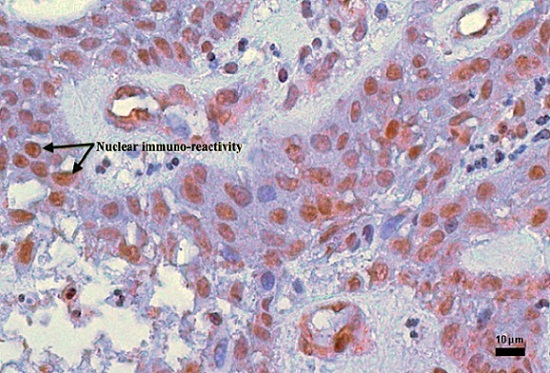
Shows plexiform ameloblastoma with MDM2 weak (+) nuclear positivity for ameloblast (X100)

Approximately 67.9% of our study population was in the 3^rd^ decade of life and there was equal gender distribution. For the islands of odontogenic epithelium reacting to PTCH, both the ameloblast like cells and the stellate reticulum-like cells expressed different degrees of cytoplasmic positivity (Figure 1, Figure 2, Figure 3). In the ameloblast cell region only one case was found not to show expression for PTCH while moderate and strong expressions together constituted 78.6% of the cases (Table 1). Approximately 60.7% of the stellate reticulum showed either moderate or strong expression of PTCH (Table 1). Nine (75%) out of 12 plexiform ameloblastoma had moderate to strong PTCH expression in the ameloblast and stellate reticulum-like region while all the follicular ameloblastoma expressed PTCH moderately in the ameloblast region (Table 2). Only 3 (10.7%) cases expressed MDM2, all showing weak immunoreactivity (Figure 4); two of them were males with plexiform ameloblastoma while the third case was a female with cystic ameloblastoma. All the 3 patients were in the 3^rd^ decade of life.

**Table 1:** PTCH expression in ameloblast and stellate reticulum

PTCH expression	Frequency (%) None	Weak	Moderate	Strong	Total
Ameloblast	1 (3.6)	5 (17.9)	11 (39.3)	11 (39.3)	28 (100)
Stellate Reticulum	2 (7.1)	9 (32.1)	8 (28.6)	9 (32.1)	28 (100)

**Table 2:** PTCH expression in variants of ameloblastoma

	Plexiform		Follicular		Cystic		Hemangiomatous	
**PTCH expression**	A	S	A	S	A	S	A	S
None	0	1	0	0	1	1	0	0
Weak	3	2	0	2	1	5	1	0
Moderate	5	3	4	1	2	3	0	1
Strong	4	6	0	1	6	1	1	1
Total	12		4		10		2	
A= Ameloblast, S= Stellate Reticulum								

## Discussion

Our immunohistochemical based study found moderate to strong expression of PTCH in ameloblastoma. A study by DeVilliers et al [10] that determined the microgenomic profile of ameloblastoma via several molecular methods reported that most members of the Sonic Hedgehog pathway were under-expressed except for PTCH that was over-expressed by two-folds. They additionally validated protein expression in their study by immunohistochemistry. Heikinheimo et al [5] however documented an under-expression of PTCH and other Sonic Hedgehog pathway members via several molecular methods. DeVilliers et al [10] had earlier suggested that the over-expression of PTCH could be used for chemotherapeutics and from our findings we support this proposition in Nigerian ameloblastoma cases. Shiori et al [17] recently reported that the proliferation of the ameloblastoma AM-1 cell line was significantly inhibited in the presence of SHH neutralizing antibody and cyclopamine. Keratocystic odontogenic tumour, which is in the same odontogenic tumor group as ameloblastoma, and also has PTCH dysfunction implicated in its pathogenesis has been shown to respond significantly to cyclopamine in a dose-dependent manner by targeting the SHH pathway [18]. Strong expression for PTCH in ameloblast-like region was noted in 60% of cystic ameloblastoma. This is comparable to the expression in other neoplastic odontogenic cysts [19] and may also support the report of Rosenstein et al [20] who documented that the percentage of cells in the cell cycle was higher in cystic ameloblastoma compared to the solid variant. Thus, although the cystic ameloblastoma may appear innocuous, it actually has higher biologic behavior at the ameloblast cellular level and may thus have a higher expression of PTCH which is a gene related to proliferation. Hemangiomatous ameloblastoma is a rare variant and although there was moderate to strong PTCH expression in the ameloblast and stellate reticulum-like regions of these cases, they are not sufficient for prognostic or therapeutic prediction.

MDM2 negatively inhibits p53 and hence the over-expression of MDM2 will greatly suppress the tumor suppressor activity of p53 [21]. MDM2 expression in this study was very poor and this may suggest that in Nigerian patients with ameloblastoma, MDM2 does not play a significant role in p53 inhibition. Other studies in non-Nigerian patients have shown that p53 in ameloblastoma are mostly suppressed by MDM2 and not mutated. Hence, if MDM2 is detached from them, they can resume their tumor suppressor action. However, our study raises the question of primary mutation in the p53 tumor suppressor protein in Nigerian ameloblastoma cases and not p53 inhibition by MDM2. Further studies are needed to establish the status of the p53 gene in Nigerian ameloblastoma cases. A study from Malaysia reported a frame-shift type mutation of p53 in ameloblastoma at exon 4 and 7 [21]. They then suggested that this mutation could serve for potential therapeutics. Anti-p53 agents could reduce the size of large and or unresectable tumors close to vital structures [21].

## Conclusion

The significance of our study therefore is that it supports, in theory, anti-PTCH/SHH chemotherapeutics for Nigerian ameloblastoma cases and also infers the possible additional use of anti-p53 agents.

## References

[1] Reichart PA, Philipsen HP, Sonner S (1995). Ameloblastoma: biological profile of 3677 cases. Eur J Cancer B Oral Oncol..

[2] Arotiba JT, Ogunbiyi JO, Obiechina AE (1997). Odontogenic tumours: a 15-year review from Ibadan, Nigeria. Br J Oral Maxillofac Surg..

[3] Chidzonga MM, Lopez VM, Alverez AP (1996). Odontogenic tumours; analysis of 148 cases in Zimbabwe. Cent Afr J Med..

[4] Ladeinde AL, Ajayi OF, Ogunlewe MO, Adeyemo WL, Arotiba GT, Bamgbose BO, Akinwande JA (2005). Odontogenic tumors: a retrospective analysis of 319 cases in a Nigerian teaching hospital. Oral Surg Oral Med Oral Pathol Oral Radiol Endod..

[5] Heikinheimo K, Jee KJ, Niini T, Aalto Y, Happonen RP, Leivo I, Knuutila S (2002). Gene expression profiling of ameloblastoma and human tooth germ by means of a cDNA microarray. J Dent Res..

[6] Kumamoto H, Ooya K (2008). Immunohistochemical detection of BH3-only proteins in ameloblastic tumors. Oral Dis..

[7] Berman DM, Karhadkar SS, Hallahan AR, Pritchard JI, Eberhart CG, Watkins DN, Chen JK, Cooper MK, Taipale J, Olson JM, Beachy PA (2002). Medulloblastoma growth inhibition by hedgehog pathway blockade. Science..

[8] Yanai K, Nagai S, Wada J, Yamanaka N, Nakamura M, Torata N, Noshiro H, Tsuneyoshi M, Tanaka M, Katano M (2007). Hedgehog signaling pathway is a possible therapeutic target for gastric cancer. J Surg Oncol..

[9] Koyama E, Wu C, Shimo T, Iwamoto M, Ohmori T, Kurisu K, Ookura T, Bashir MM, Abrams WR, Tucker T, Pacifici M (2001). Development of stratum intermedium and its role as a Sonic hedgehog signalling structure during odontogenesis. Dev Dyn..

[10] DeVilliers P, Suggs C, Simmons D, Murrah V, Wright JT (2011). Microgenomics of Ameloblastoma. J Dent Res..

[11] Nishimaki H, Kasai K, Kozaki K, Takeo T, Ikeda H, Saga S, Nitta M, Itoh G (2004). A role of activated Sonic hedgehog signalling for the cellular proliferation of oral squamous cell carcinoma cell line. Biochem Biophys Res Commun..

[12] Mukherjee S, Frolova N, Sadlonova A, Novak Z, Steg A, Page GP, Welch DR, Lobo-Ruppert SM, Johnson MR, Frost AR (2006). Hedgehog signalling and response to cyclopamine differ in epithelial and stromal cells in benign breast and breast cancer. Cancer Biol Ther..

[13] Freedman DA, Wu L, Levine AJ (1999). Functions of the MDM2 oncoprotein. Cell Mol Life Sci..

[14] Asslaber D, Pinon JD, Seyfried J, Desch P, Stocher M, Tinhofer I, Egle A, Merkel O, Grell R (2010). microRNA-34a expression correlates with MDM2 SNP 309 polymorphism and treatment free survival in chronic lymphocytic leukemia. Blood..

[15] Krishna A, Kaveri H, Naveen Kumar R K, Kumaraswamy K L, Shylaja S, Murthy S (2012). Overexpression of MDM2 protein in ameloblastomas as compared to adenomatoid odontogenic tumor. J Can Res Ther..

[16] Sinicrope FA, Ruan SB, Cleary KR, Stephens LC, Lee JJ, Levin B (1995). bcl-2 and p53 onco-protein expression during colorectal tumorigenesis. Cancer Res..

[17] Shiori K, Takeshi M, Yu N, Shintaro K, Yuichi G, Ryota M, Seiji N (2013). Anti-apoptotic role of the sonic hedgehog signaling pathway in the proliferation of ameloblastoma. International Journal of Oncology..

[18] Ren C, Amm HM, DeVilliers P, Wu Y, Deatherage JR, Liu Z, MacDougall M (2012). Targeting the sonic hedgehog pathway in keratocystic odontogenic tumor. J Biol Chem..

[19] Vered M, Peleg O, Taicher S, Buchner A (2009). The immunoprofile of odontogenic keratocyst (keratocystic odontogenic tumor) that includes expression of PTCH, SMO, GLI-1 and bcl-2 is similar to ameloblastoma but different from odontogenic cysts. J Oral Pathol Med..

[20] Rosenstein T, Pogrel MA, Smith RA, Regezi JA (2001). Cystic ameloblastoma behavior and treatment of 21 cases:; discussion 1316-8. J Oral Maxillofac Surg..

[21] Al-Salihi KA, Li LY, Azlina A (2006). P53 gene mutation and protein expression in ameloblastomas. Braz J Oral Sci..

